# The associations of dyadic coping strategies with caregivers’ willingness to care and burden: A weekly diary study

**DOI:** 10.1177/13591053231223838

**Published:** 2024-01-10

**Authors:** Giulia Ferraris, Pierre Gérain, Mikołaj Zarzycki, Saif Elayan, Val Morrison, Robbert Sanderman, Mariët Hagedoorn

**Affiliations:** 1 University Medical Center Groningen, University of Groningen, The Netherlands; 2 Vrije Universiteit Brussel, Belgium; 3 Liverpool Hope University, UK; 4 Bangor University, UK

**Keywords:** between-differences, burden, caregiving, diary, dyadic coping, willingness to care, within-processes

## Abstract

This weekly diary study investigated associations of weekly dyadic coping strategies with caregivers’ willingness to care and burden. Multilevel modelling was applied to assess between- and within-person associations for 24 consecutive weeks in 955 caregivers. Greater willingness to care was reported in weeks when caregivers used more collaborative (*b* = 0.26, *p* < 0.001) and supportive (*b* = 0.30, *p* < 0.001) strategies, whereas uninvolved coping was associated with lower willingness to care (*b* = −0.44, *p* < 0.001). Using collaborative coping strategies was associated with lower weekly burden (*b* = −0.13, *p* < 0.001). A greater burden was reported in weeks when caregivers used more uninvolved (*b* = 0.19, *p* < 0.001) and controlling (*b* = 0.13, *p* < 0.001) coping strategies. A full understanding of whether caregivers’ willingness to care and burden may be improved owing to weekly dyadic coping is essential for developing timely support for caregivers.

Chronic illnesses, such as heart disease, stroke, cancer and dementia, significantly impact the daily lives of both patients and their informal caregivers, who are often family members or friends (Revenson et al., 2016). Informal caregivers, hereafter referred to as caregivers, play a crucial role in managing illness-related stressors and providing support. Effective coping strategies employed by caregivers can enhance their willingness to continue caregiving and reduce caregiver burden, whereas ineffective coping may lead to reduced willingness and burnout (Hawken et al., 2018). However, despite the importance of these processes, little is known about how caregivers’ daily dyadic coping interactions with care recipients, notably dyadic coping, are related to fluctuations in caregiving willingness to care and burden. Therefore, this intensive longitudinal study (i.e. weekly diary study) aims to explore whether and how dyadic coping, caregiver willingness and caregiver burden occur in the daily lives of caregivers providing care to their spouse, older parent, sibling or any other family member or friend.

Although the developmental-contextual model refers to dyadic coping as a process that ‘sequentially unfolds in more discrete time moments across a conversation or over days’ (Berg and Upchurch, 2007), existing research on dyadic coping in chronic diseases has primarily focused on long-term effects and between-person differences, limiting our understanding of daily fluctuations in dyadic coping. Specifically, traditional longitudinal studies provide information on how people differ from each other when using positive (e.g. supportive, empathetic, collaborative) versus negative (e.g. hostile, ambivalent, critical) dyadic coping strategies in terms of increased or decreased relationship quality over a long interval of time (e.g. over 1 year) (Bodenmann et al., 2006; Falconier and Kuhn, 2019). Such literature on between-person differences risks obscuring weekly or daily processes that may occur when caregivers cope with their care recipients’ needs. Diary methods involving repeated assessments over a short period (e.g. daily measurements for consecutive days or weeks) are essential for gaining deeper insights into individuals’ lived experiences. These methods allow us to move beyond between-level differences and explore fluctuations in dyadic coping within each person (i.e. within-person variability). Additionally, diary studies are particularly valuable for examining the connections between daily stressors and wellbeing (Gérain et al., 2023). This approach might facilitate the investigation of whether the adoption of specific dyadic coping strategies is effective for individuals by comparing days or weeks when the strategy is used more frequently to weeks when it is used less (Berg and Upchurch, 2007; Bolger and Laurenceau, 2013).

Early diary studies showed substantial day-to-day variability in collaborative dyadic coping strategies among couples dealing with prostate cancer (Berg et al., 2008b), parents and adolescents with diabetes (Berg et al., 2008a) and couples dealing with diabetes (Zajdel et al., 2018). Other diary research has focused on shared illness appraisal and collaboration by examining links between communal coping, mood, support exchange and illness adjustment (Jones et al., 2023; Zajdel et al., 2023). However, previous literature on diary studies exploring dyadic coping processes has been limited (Weitkamp and Bodenmann, 2022) and tends to overlook the fact that caregivers can frequently switch between or use multiple strategies while providing daily care (Badr et al., 2010; Kleiboer et al., 2006; Kroemeke and Sobczyk-Kruszelnicka, 2019). For instance, daily control and support were found to co-occur in caregivers of care recipients with diabetes (August et al., 2013). It is not clear from these studies, however, whether dyadic coping fluctuates over a short interval of time and whether alternating different strategies might be functional and in line with changes in the illness.

To address this research gap, we present an intensive longitudinal study that utilizes weekly diary assessments to investigate the fluctuations of various dyadic coping strategies (e.g. uninvolved, controlling, supportive and collaborative) within caregivers over time. Moreover, we aim to establish a direct link between these coping strategies and caregivers’ willingness to care and burden. This approach aligns with the developmental-contextual model of dyadic coping (Berg and Upchurch, 2007), which suggests that caregivers may use multiple coping strategies as they manage chronic illnesses at any given time and also over time. Caregivers might transition from *uninvolved* strategies, when each dyad member copes relatively independently from one another (e.g. limiting discussions), to *controlling* strategies, when one dyad member tries to direct or dominate the coping efforts of the other (e.g. telling the other person what to do), to *supportive* strategies, providing emotional and/or instrumental support or assistance to the other, and to *collaborative* ones when both dyad members work together to manage the problem (e.g. they may sit down together and discuss treatment options) (Berg and Upchurch, 2007).

By studying the fluctuations in dyadic coping strategies, it is also possible to investigate whether changes in these coping strategies are associated with changes in caregivers’ willingness to continue providing care and the level of burden they experience. Willingness to care and burden are relevant caregiving outcomes but hardly investigated in association with dyadic coping. The majority of studies mainly explored dyadic coping associated with the care recipient’s adjustment to the illness (Shi et al., 2021) and/or relationship functioning, such as intimacy (Belcher et al., 2011; Otto et al., 2015) and relationship satisfaction (Langer et al., 2009). Little is known about whether and how dyadic coping might help caregivers find adaptive strategies to overcome difficulties and remain willing to care and alleviate burden from week to week (Burridge et al., 2007; Ferraris et al., 2023b; Zarzycki and Morrison, 2021). Notably, caregivers are at increased risk for negative outcomes such as poor motivation outcomes (Zarzycki et al., 2023) and burden or depression (Kurtz et al., 2004). Exploring caregivers’ willingness to care and burden is crucial due to the ‘Care Gap challenge’ – the widening disparity between caregiving demand and available caregivers (de Jong et al., 2022). Identifying potential drivers (e.g. dyadic coping) that maintain caregiver willingness and reduce burden, will help to ensure sustainable and quality care while informing support policies and interventions. Indeed, understanding the role of daily variations in caregiving experiences is essential for identifying potential interventions and support systems that can mitigate caregiver burden and maintain their willingness to provide care effectively.

Lastly, there is a need to explore caregiving experiences in dyads other than romantic partners. Most dyadic coping literature so far has been limited to couples (Falconier and Kuhn, 2019). Indeed, romantic partners are often the first to provide tangible assistance and support for ill partners (Revenson et al., 2016). However, there are other types of caregiving relationships, such as adult children and older parents, siblings, friends (Colombo et al., 2011). Given the nature of romantic relationships, collaboration is expected to be a fundamental resource fostering reciprocation and wellbeing for the couple. Whilst in non-spousal relationships (e.g. adult children and older parents), role differentiation suggests that collaboration might not always be desired by caregivers. However, very little is known about dyadic coping in dyads other than spousal ones (Ferraris et al., 2022). For this reason, of particular interest for the current study is to explore whether weekly associations of dyadic coping, willingness to care and burden might differ between different relationship types.

The current study is based on a previous publication on the same sample study that found caregivers’ willingness to care to decrease over time and to fluctuate from week to week (Ferraris et al., 2023b). The first aim is to explore the associations between dyadic coping strategies and outcomes of willingness and burden. In this we will explore whether caregivers who use collaborative and supportive strategies are more willing to care and less burdened than those caregivers using uninvolved and controlling strategies (between-differences associations), and also whether on weeks when caregivers use collaborative and supportive strategies, they themselves are more willing and less burdened than when using uninvolved and controlling strategies (within-processes associations). A secondary aim is to test the moderating role of caregiving relationship type (i.e. spousal vs non-spousal), exploring whether associations between dyadic coping and willingness/burden are moderated by this factor.

## Methods

### Procedure and participants

This weekly diary study includes 24 weekly repeated questionnaires over 6 months from caregivers participating in the ENTWINE iCohort study of caregiving conducted in nine countries: Germany, Greece, Ireland, Israel, Italy, the Netherlands, Poland, Sweden and the United Kingdom. Full details of the ENTWINE iCohort can be found in the published protocol (Morrison et al., 2022) and in a previous publication (Ferraris et al., 2023a). This study was approved by all participating countries (see Morrison et al., 2022) and the primary full approval was obtained from the Institutional Review Board (or Ethics Committee) of Bangor University for non-clinical recruitment and NHS Research Ethics and Governance Committee for clinical site recruitment (protocol code 20/WA/0006, January and June 2020, respectively). All participants signed an electronic informed consent form prior to participation.

Caregivers were recruited online. To be included, caregivers had to be 18 years old or older and provide care for a family member or a friend with a chronic health condition, disability or any other care need. Exclusion criteria were not having access to the Internet and not having the cognitive capacity to complete the questionnaires online. Participants were asked to complete 24 consecutive weekly questionnaires, accessible via a computer, laptop or any other smart device (e.g. smartphones), once per week at the same time, in the afternoon (Ferraris et al., 2023b). Of the 1872 caregivers enrolled in the iCohort study, 955 (51%) agreed to participate in this weekly diary study. Participants received reminders during the week either to motivate them to stay involved in the study or to thank them for their participation. After four consecutive missing weekly diary questionnaires, participants were automatically removed from the diary study after being notified by email. A total of 22,920 diary questionnaires (955 caregivers × 24 weeks) was expected. However, 459 (48%) caregivers completed 11,016 diary questionnaires (five or fewer weeks), 138 (14%) caregivers completed 3312 diary questionnaires (between 6 and 12 weeks) and 358 (38%) caregivers completed 8592 diary questionnaires (more than12 weeks). The highest drop-out rates were registered after the first diary entry (22%); thereafter, the drop-out rate slowly declined during the diary period. Given that multilevel modelling using restricted maximum likelihood estimation is robust to missing data, we included all participants who completed one or more weeks of diary data (Singer and Willett, 2003). The sensitivity analyses with the different groups (i.e. caregivers who completed five or fewer, between 6 and 12 and more than 12 weekly questionnaires) did not show differences in the pattern of results. Results of sensitivity analyses are reported in Table 2.

## Baseline measures

### Demographics and caregiving context

Self-reported caregiver age, gender, education, relationship status, employment, care recipient’s age, gender, type and duration of health conditions were assessed by self-report in the baseline questionnaire. Caregivers were asked to complete measures concerning their main care recipients (e.g. care recipient’s health and age) and their care context (e.g. whether the caregiver presented one or more health conditions or not themselves, the intensity of care, and co-residency status of the caregiver and the care recipient).

### Relationship type

Caregivers were asked to indicate their relationship type with the care recipient, which, for the current study, was dichotomized into spousal (including spouses and partners) versus non-spousal caregivers (including adult children, parents, siblings and friends).

## Weekly measures

### Dyadic coping strategies

Based on the Developmental-Contextual Model of Dyadic Coping (Berg and Upchurch, 2007), items were developed from a previous daily diary study (Berg et al., 2008a). Each dyadic coping strategy was assessed with three items, chosen and adapted to the weekly context (e.g. referring to ‘this week’). Specifically, every week, caregivers reported on the degree to which they had been supportive (*I have listened to my loved one*), collaborative (*My loved one and I have solved caregiving issues together*), uninvolved (*I have avoided my loved one*) and controlling (*I have told my loved one what to do*) towards their care recipients. Responses ranged from 0 (*not at all*) to 4 (*extremely*). Higher mean scores indicate more frequent use of dyadic coping strategies of any type, each week.

### Willingness to care

Caregivers’ weekly momentary willingness to care was assessed with one item, using a 10-point Likert scale from 1 (*not all*) to 10 (*extremely*), based on the Willingness to care Scale (Abell, 2001). The item was adapted to measure caregivers’ willingness to care at that specific moment of that week (*How willing are you to look after your loved one’s needs right now?*). Higher mean scores indicate a greater weekly willingness to care.

### Caregiver burden

Caregivers reported their weekly momentary level of burden with one item (*Do you feel that caring for your loved one is a burden right now?*), ranging from 1 (*not at all*) to 5 (*extremely*), based on the Short Form Zarit Burden Interview (Bédard et al., 2001). Higher scores indicate a greater feeling of weekly burden.

The reliability indicators, which indicate the overall consistency of items across time and individuals, showed very high reliabilities for all the weekly scales on the between-person level (R_KRN_) ranging from 0.98 to 0.95 and satisfactory on the within-person level (R_CN_) ranging from 0.47 to 0.72 (see Table S1). The Cronbach alphas at each measurement point are in the supplementary materials (Table S2).

## Statistical analysis

Multilevel modelling was conducted in IBM SPSS statistics 28 to analyse weekly diary questionnaire data (within level 1: time) nested within caregivers (between level 2: individuals). Multilevel models allow for the disaggregation of between-person differences and within-person fluctuations in the outcomes (i.e. willingness to care and burden). However, they cannot automatically distinguish the two levels for the predictors (i.e. dyadic coping strategies). Thus, to prevent this confounding of levels from occurring, the diary dyadic coping measures (level 1 predictors) were person-mean centred (i.e. participants’ mean scores were subtracted from their raw score at each assessment point) and were also included as grand-mean centred variables (i.e. the group mean was subtracted from each individual score) (Bolger and Laurenceau, 2013).

Descriptive analyses were carried out (i.e. means and standard deviations for continuous variables and frequencies and percentages for categorical variables) to describe the sample (Table 1). Before testing the three research questions, we examined the means, the standard deviations and the between-person correlations of all the variables (Table S1).

**Table 1.** Sample characteristics and descriptive of caregivers.

Caregivers	*N* (%)	*M* (SD)
Age		56.72 (11.27)
Gender		
Female	854 (89.4%)	
Male	96 (10.1%)	
Other	5 (0.6%)	
Education		
Primary	10 (1.0%)	
Secondary	138 (14.5%)	
Post-secondary vocational	376 (39.4%)	
Post-secondary academic	416 (43.6%)	
Other	15 (1.6%)	
Relationship status		
Single	137 (14.3%)	
Married	663 (69.4%)	
Divorced	95 (9.9%)	
Widowed	30 (3.1%)	
Other	30 (3.1%)	
CG health conditions (yes)	440 (46.1%)	
Currently employed (yes)	463 (48.5%)	
Intensity of care		43.14 (36.80)
Care recipients’ information given by the CG		
CR age		68.69 (19.86)
CR gender		
Female	465 (48.7%)	
Male	481 (50.4%)	
Other	9 (0.9%)	
Relation of the CR to the CG		
Spouse/partner	355 (37.2%)	
Parent/parent-in-law	403 (42.2%)	
Daughter/son	106 (11.1%)	
Another family member	52 (5.4%)	
Non-relative member	39 (4.1%)	

Preliminary analyses allowed us to explore the degree of variability in all the variables (i.e. dyadic coping strategies, willingness to care and burden) attributable to the between- and within-person levels. Intercept-only models (i.e. no predictor variables were included) were estimated. From this model, we were able to calculate the intraclass correlation coefficients (ICCs) to decompose the proportion of variance that varied on average from person to person (i.e. at level 2) and that also varied within each person over time (i.e. at level 1).

For the first research question two separate multilevel models (model 1 with willingness to care as the outcome, model 2 with burden as the outcome), including all person-mean and grand-mean centred dyadic coping strategies as predictor variables, were run. Time, centred in a way that zero represents the first diary week and one the change over the 24-week diary period, was included as a predictor in all the models (Bolger and Laurenceau, 2013). Finally, for the second research question the two multilevel models were re-run in interaction with relationship type (spousal vs non-spousal caregivers) to estimate whether between and within-subject associations differed for spousal and non-spousal caregivers.

Moreover, we controlled for the caregiver’s age (*r_willingness_* = 0.05, *p* < 0.001), care recipient’s age (*r_willingness_* = −0.08, *p* < 0.001; *r_burden_* = 0.13, *p* < 0.001), caregiver’s gender (*t*_(9740,1) *willingness*_ = −9.16, *p* < 0.001; *t*_(9740,1) *burden*_ = 3.86, *p* < 0.001), care recipient’s gender (*t*_(9740,1) *willingness*_ = 3.31, *p* < 0.001; *t*_(9740,1) *burden*_ = 7.41, *p* < 0.001), care recipient’s illness condition (*F*_(9740,1) *willingness*_ = 71.71, *p* < 0.001; *F*_(9740,1) *burden*_ = 235.21, *p* < 0.001), caregiver’s education (*F*_(9740,1) *willingness*_ = 30.33, *p* < 0.001; *F*_(9740,1) *burden*_ = 25.90, *p* < 0.001) and caregiver’s relationship status (*F*_(9740,1) *burden*_ = 64.70, *p* < 0.001) because the variables of interest have been found to be associated with either willingness to care and/or burden. An unstructured variance-covariance matrix was used to accommodate the nested nature of the data. Moreover, in all the models, we controlled for autocorrelation of within-level residuals (Bolger and Laurenceau, 2013).

## Results

The sample consisted of 955 caregivers with an average age of 56.7 years (range 18–88) and mostly female (89.4%), while about half of the care recipients were male (50.4%) with an average age of 68.6. Caregivers were mainly non-spousal (62.8%; *n* = 600), and the largest group provided care for a parent (42.2%; *n* = 403). In total, 38.8% of the caregivers reported providing care to a person with a physical impairment only, 26% to a person with neurological/mental impairments and 33.7% to people with comorbidity of physical and neurological conditions, providing on average 43.1 hours of care per week. Additional sample characteristics and descriptive information of caregivers are reported in Table 1 and in another previous publication (Elayan et al., in press).

## Preliminary results: Variability of dyadic coping strategies, willingness to care and burden

Overall, results showed that half or more of the variabilities of dyadic coping strategies and willingness/burden originated from between-person differences. A remaining substantial variability also occurred in within-person fluctuations and residual errors. Specifically, 45% of the variation in uninvolved, 40% in supportive, 28% in collaborative and 28% in controlling dyadic coping strategies were in weekly fluctuations and errors (within-level variability). Willingness to care and burden showed relatively low variability in weekly fluctuations, respectively 32% and 35%. ICCs are displayed in Table S1. On average, caregivers most frequently reported using supportive coping strategies followed by controlling, collaborative and uninvolved strategies. Also, caregivers had relatively high levels of willingness to care and burden (see means and SDs in Table S1).

## Research question 1: Associations of dyadic coping strategies, willingness to care and burden

Results of the multilevel model with willingness to care and burden are shown in Table 2. Fixed effects estimated willingness to care (intercept *b* = 8.31, *p* < 0.000) and burden (intercept *b* = 2.29, *p* < 0.000) for a typical caregiver and showed a significant linear decrease over time in willingness to care (slope *b* = −0.65, *p* < 0.001) but not significant linear time trends for burden (slope *b* = 0.05, *p* = 0.092). All the coefficients can be interpreted in the same manner as regression coefficients. For example, an increase of 1 unit on time (number of weeks) would translate into a 0.65 unit decrease in willingness to care.

**Table 2.** Associations of dyadic coping strategies, willingness to care and burden.

Fixed effects (intercept, slopes)	Willingness to care					Burden				
	*b* (SE)	*t*_(954)_	*p*	CI_95%_		*b* (SE)	*t*_(954)_	*p*	CI_95%_	
				Lower	Upper				Lower	Upper
Intercept	8.31 (0.50)	16.35	<0.001	7.31	9.31	2.29 (0.26)	8.54	<0.001	1.77	2.82
Time slope (week 0–23)	−0.65 (0.06)	−10.01	<0.001	−0.78	−0.52	0.05 (0.03)	1.68	0.092	−0.00	0.12
*Between-person associations*										
Collaborative DC	0.40 (0.06)	5.99	<0.001	0.27	0.53	−0.29 (0.03)	−8.40	<0.001	−0.36	−0.22
Supportive DC	0.52 (0.09)	5.54	<0.001	0.33	0.70	0.05 (0.04)	1.03	0.303	−0.04	0.14
Uninvolved DC	−1.27 (0.09)	−13.26	<0.001	−1.46	−1.08	0.51 (0.05)	10.28	<0.001	0.41	0.61
Controlling DC	0.08 (0.07)	1.20	0.227	−0.05	0.22	0.34 (0.03)	9.40	<0.001	0.27	0.42
*Within-person associations*										
Collaborative DC	0.26 (0.02)	8.89	<0.001	0.20	0.32	−0.13 (0.07)	−7.74	<0.001	−0.17	−0.10
Supportive DC	0.30 (0.03)	8.56	<0.001	0.23	0.37	0.02 (0.01)	1.25	0.209	−0.01	0.05
Uninvolved DC	−0.44 (0.04)	−10.80	<0.001	−0.52	−0.36	0.19 (0.01)	9.90	<0.001	0.15	0.23
Controlling DC	−0.02 (0.03)	−0.76	0.446	−0.08	0.03	0.13 (0.01)	7.45	<0.001	0.09	0.17
*Covariates*										
CG age	0.01 (0.00)	1.83	0.067	−0.00	0.02	0.05 (0.00)	1.77	0.010	0.01	0.02
CR age	−0.00 (0.00)	−1.65	0.099	−0.01	0.00	0.00 (0.00)	1.94	0.052	−0.00	0.00
CG gender	0.62 (0.20)	3.09	0.002	0.22	1.01	−0.11 (0.10)	−1.13	0.258	−0.32	0.08
CR gender	0.04 (0.12)	0.35	0.742	−0.20	0.29	0.07 (0.06)	1.16	0.245	−0.05	0.20
Relationship status	0.76 (0.06)	3.56	0.002	−0.45	1.50	−0.01 (0.03)	−0.48	0.629	−0.09	0.05
CR illness condition	−0.05 (0.07)	−0.83	0.405	−0.20	0.08	0.04 (0.03)	1.26	0.205	−0.02	0.12
Education	−0.27 (0.07)	−3.47	<0.001	−0.42	−0.11	0.10 (0.04)	2.58	0.010	0.02	0.18
Random effects ((co) variances)	*b* (SE)	*z*	*p*	CI_95%_		*b* (SE)	*z*	*p*	CI_95%_	
				Lower	Upper				Lower	Upper
*Between-level*										
Collaborative intercept^a^	0.80 (0.15)	17.58	<0.001	2.51	3.13	0.70 (0.04)	17.25	<0.001	0.62	0.78
Collaborative slope	0.10 (0.02)	3.96	<0.001	0.06	0.16	0.03(0.00)	4.20	<0.001	0.02	0.06
Covariance collaborative intercept and time	−0.38 (0.06)	−6.39	<0.001	−0.50	−0.26	−0.00 (0.01)	−0.25	0.799	−0.03	0.02
Supportive intercept	−0.18 (0.06)	−2.92	0.004	−0.31	−0.06	0.02 (0.01)	1.32	0.185	−0.01	0.05
Supportive slope	0.19 (0.03)	5.25	<0.001	0.13	0.27	0.03(0.00)	4.15	<0.001	0.02	0.05
Covariance supportive intercept and time	0.03 (0.02)	1.34	0.180	−0.01	0.07	0.00 (0.00)	0.56	0.571	−0.00	0.01
Uninvolved intercept	0.02 (0.07)	0.40	0.685	−0.11	0.16	−0.03 (0.01)	−1.88	0.060	−0.07	0.00
Uninvolved slope	0.31 (0.05)	6.03	<0.001	0.23	0.44	0.04 (0.01)	3.84	<0.001	0.02	0.06
Covariance uninvolved intercept and time	−0.01 (0.02)	−0.59	0.552	−0.07	0.03	0.00 (0.00)	0.17	0.865	−0.01	0.01
Controlling intercept	0.01 (0.05)	0.28	0.777	−0.09	0.12	0.00 (0.01)	0.26	0.794	−0.02	0.03
Controlling slope	0.10 (0.02)	3.88	<0.001	0.06	0.18	0.03 (0.00)	3.55	<0.001	0.02	0.06
Covariance controlling intercept and time	0.01 (0.02)	0.48	0.626	−0.03	0.05	−0.00 (0.00)	−0.63	0.526	−0.01	0.00
*Within-level*										
Residual	1.69 (0.03)	53.56	0.000	1.63	1.75	0.52 (0.00)	55.62	0.000	0.50	0.54
Autocorrelation	0.24 (0.01)	18.35	<0.001	0.22	0.27	0.19 (0.01)	14.34	<0.001	0.16	0.21

Sensitivity analyses results: Group 1 (caregivers who completed five or fewer weekly questionnaires): Within-person association between ‘Collaborative DC’ and ‘Willingness to Care’ (*b* = 0.28, SE = 0.03), and between ‘Collaborative DC’ and ‘Burden’ (*b* = −0.15, SE = 0.02). Group 2 (caregivers who completed between 6 and 12 weekly questionnaires): Within-person association between ‘Collaborative DC’ and ‘Willingness to Care’ (*b* = 0.27, SE = 0.02), and between ‘Collaborative DC’ and ‘Burden’ (*b* = −0.14, SE = 0.02). Group 3 (caregivers who completed more than 12 weekly questionnaires): Within-person association between ‘Collaborative DC’ and ‘Willingness to Care’ (*b* = 0.29, SE = 0.03), and between ‘Collaborative DC’ and ‘Burden’ (*b* = −0.16, SE = 0.02).

CG: caregiver; CR: care recipient; DC: dyadic coping strategies.

a Expressed as standard deviation, the variation for the collaborative intercept, when willingness to care is 0, is √0.80 = 0.8 units, which implies that 95% of the population varies between ±2 × 0.8 = ±1.6 units of the typical collaborative value, which is a range of intercepts of 0.40 ± 1.6 = −1.2 to 2. The same procedure must be applied for all the predicted values.

Estimates of fixed effects of the between- and within-person slopes for dyadic coping strategies showed significant between- and within-person associations between three out of four dyadic coping strategies and willingness to care and burden. Specifically, on a between-person level, caregivers who reported greater *collaborative* dyadic coping strategies than the sample average also reported a greater willingness to care (*b* = 0.40, *p* < 0.001) and lower burden across time (*b* = −0.29, *p* < 0.001). Also, caregivers who reported greater *supportive* dyadic coping strategies than others reported greater willingness to care (*b* = 0.52, *p* < 0.001). Caregivers who reported greater *uninvolved* dyadic coping strategies than the average of the sample reported lower willingness to care (*b* = −1.27, *p* < 0.001) and greater burden across time (*b* = 0.51, *p* < 0.001). Also, caregivers who reported greater *controlling* dyadic coping strategies than others reported greater burden across time (*b* = 0.34, *p* < 0.001).

Similar effects were reported on a within-person level of analysis. On weeks when caregivers showed more *collaborative* dyadic coping strategies than their own average, they also reported a greater willingness to care (*b* = 0.26, *p* < 0.001) and lower burden (*b* = −0.13, *p* < 0.001). Similarly, on weeks when caregivers showed more *supportive* dyadic coping strategies than their own average, they also reported a greater willingness to care (*b* = 0.30, *p* < 0.001). In contrast, on weeks when caregivers showed greater *uninvolved* dyadic coping strategies than their own average, they reported lower willingness to care (*b* = −0.44, *p* < 0.001) and greater burden (*b* = 0.19, *p* < 0.001) and on weeks when caregivers showed greater *controlling* dyadic coping strategies than their own average, they reported greater burden (*b* = 0.13, *p* < 0.001).

Estimates of random effects indicated variances at two levels of analysis. At the between-level, which looks at differences among caregivers, there were variations in the initial starting points (intercepts) and the slopes (rate of change) concerning the associations between dyadic coping strategies and willingness to care/burden. In other words, different caregivers showed different patterns of how their coping strategies related to their willingness to care and the burden experienced. Covariance between intercept and slope was significant only for collaborative dyadic coping strategies; caregivers who initially reported higher or lower levels of willingness to care (larger or smaller intercepts) tended to experience a corresponding increase or decrease in their willingness to care over time (larger or smaller slopes) when they employed collaborative dyadic coping strategies. This suggests that the use of collaborative coping strategies had an impact on how caregivers’ willingness to care changed as time progressed. On a within-level, an estimate of the size of the residuals’ variance was significant for willingness to care (*b* = 1.69, *p* < 0.001) as well as for burden (*b* = 0.52, *p* < 0.001), meaning that the variability in caregivers’ willingness to care and burden is not due to chance and is likely related to unmeasured or unaccounted-for factors. The lower panel of Table 2 presents numerical estimates of variability reported as variances and covariances and expressed as standard deviations in the notes (see Table 2).

## Research question 2: The moderating role of caregiving relationship type

Both between- and the within-person associations between dyadic coping, willingness and burden were not moderated by the type of relationship (spousal vs non-spousal). A complete table with dyadic coping in interaction with relationship type can be found in the Supplemental Materials (Table S3).

## Discussion

This weekly diary study demonstrated that all the dyadic coping strategies (i.e. collaborative, supportive, uninvolved and controlling) and caregiver willingness to care and burden were found to differ considerably from one caregiver to another (between-person variability) but also to fluctuate in the same caregiver from week to week (within-person variability). Associations between caregivers’ dyadic coping strategies and willingness to care/burden were found both at an individual difference level (between-persons associations) as well as at a weekly level (within-person associations). These associations did not differ based on the relationship type (spousal vs non-spousal) between caregivers and care recipients.

Both the between- and within-level associations were detected in the same model, implying that they have independent relevance to the outcomes of caregiver willingness and burden, signifying their equal importance in the context of longitudinal data analysis. On a between-person level and in line with the existing literature (Berg et al., 2008a), we found a global time trend of caregivers engaging in more collaborative and supportive efforts with the care recipients being more willing and less burdened than those caregivers who were less involved and controlling. At the same time, we found that individual caregivers’ willingness to care and burden can develop from the accumulation of their repeated weekly dyadic coping strategies, suggesting that also weekly individual variations of dyadic coping contributed to explaining variance in individual caregivers’ willingness/burden (within-level). Diary studies appear to be an ideal framework for investigating whether between-person differences are also present on a weekly/daily level (Gérain et al., 2023). Both are important, as insights into between-person differences indicate which persons might need psychological support (e.g. caregivers who perceive higher burden), while insights into within-person effects indicate which processes need to be targeted in these individuals (e.g. uninvolved and controlling strategies).

The presence of within-person associations in our results emphasizes the importance of exploring such constructs on a weekly level and points to a more dynamic account of dyadic coping processes (Bolger and Laurenceau, 2013). Specifically, our results suggested that on weeks when caregivers are more collaborative with their care recipients than usual, caregivers perceive greater willingness to care as well as less burden. This is in line with a daily diary study that underscored the everyday effect of dyadic coping in chronic illness: on days when couples reported more collaborative coping, they also felt more positive mood (Berg et al., 2008a). In the illness context, collaborative coping has been conceptualized as a resource leading to emotional benefits (Helgeson et al., 2018; Lyons et al., 1998) which may optimize task performance, compensate for a lack of skills, enhance the quality of the relationship and help regulate emotional reactions to health problems (Meegan and Berg, 2002). Thus, the beneficial effects of collaboration, in terms of increases in willingness to care and decreases in burden, are clearly supported by our findings.

In our findings, similar results as for collaboration were reported during weeks when caregivers perceived themselves to be more supportive (either instrumentally or emotionally) and more willing to care, but with no effect on burden. In other words, although caregivers were more engaged in supportive behaviours and felt more willing to provide care, their burden levels did not change during these weeks. The reasons behind this lack of effect on burden warrant further investigation. Perhaps the lack of effect on burden in the short term could be due to the specific time frame or measurement used in the study. It is possible that the effect on burden might manifest in the longer term or under different circumstances not captured during the study period. Also, caregivers who feel more supportive and willing to care may also derive a sense of fulfilment and purpose from their caregiving role (Pristavec, 2019). This sense of purpose and fulfilment might counterbalance the burden they experience, resulting in a lack of a direct effect on burden levels. On the other hand, our results highlighted how the occurrence of uninvolved and controlling strategies might be associated with episodes of negative outcomes for caregivers. On weeks when caregivers were not involved in care tasks, perhaps to reduce their own stress (August et al., 2013), they also reported decreases in their willingness to care and increases in burden. Similarly, on weeks when they were seeking to control the care recipients, this was found to be burdensome for caregivers, as constantly monitoring and attempting to regulate the care recipient’s behaviours might lead to higher burden levels, but with no effect on willingness to care. Controlling strategies may be either counterproductive in fostering willingness to care or facilitating and perhaps increasing willingness (Williams et al., 2014). Again, reasons behind this lack of effect warrant further investigation.

There are several reasons why supportive and collaborative strategies might be more beneficial than uninvolved and controlling ones. First, weekly joint coping efforts and being supportive while providing care pose the basis for a definition of chronic illness as a ‘we-disease’ (Kayser et al., 2007), especially if what Berg et al. (2008a) called an ‘interpersonal enjoyment of collaboration’ occurs. Second, given that chronic illness is experienced by both the dyad members, perceiving that both are involved in a mutual coping process, and specifically caregivers perceiving the care recipient as collaborative, might be interpreted as a sign of appreciation and love, reinforcing the caregiving role week by week (Kleiboer et al., 2006). Third, although uninvolved and control strategies permit caregivers to preserve a certain degree of self-care for their needs on some weeks, this might also prevent the caregiver from seeing collaboration opportunities with the care recipient and making use of the care recipients’ resources. Indeed, uninvolved and controlling strategies might convey the idea that the care recipient is an impaired and in-need subject, with no resources and strengths, which can, in turn, elicit caregivers’ reluctance to care and overburden themselves (Burridge et al., 2007).

The absence of a relationship-type moderator in the results indicates that the observed effects of dyadic coping might not be contingent on whether the caregivers are in romantic or other types of relationships. Collaboration and support perceptions extends beyond couples, indicating that the positive effects observed with collaborative coping and support might not be limited to romantic partners caring for each other but also apply to other caregiver-care recipient relationships, such as parent-child, sibling or friend caregiving (Ferraris et al., 2022). Further exploration of dyadic coping models in caregiving dyads beyond couples and in different contexts is needed (Revenson et al., 2016).

The findings of the current study led to a recommendation for further diary studies to explore more fully how caregivers and care recipients interact in everyday life (Bolger et al., 2003; Nezlek, 2020). Future research using repeated questionnaires over time could provide a clearer picture of when and which caregivers benefit most (and least) from dyadic coping strategies with their care recipients. Another fruitful direction for future research would be to examine daily/weekly dyadic coping processes in the context of specific phases of the illness (e.g. during making treatment decisions or after diagnosis) or specific care tasks (i.e. instrumental, nursing, emotional) to understand whether some strategies might be adaptive (or not) depending on the context, as suggested by Berg’s and Upchurch’s (2007) model. Moreover, given that appraising stress or problems as shared issues can be the starting point for collaborative coping (Berg and Upchurch, 2007), further research might evaluate and include illness appraisal as a component of dyadic coping strategies. Using a dyadic design (i.e. including both the dyad members) might shed light on the benefits of coping congruence (i.e. both dyad members using similar coping strategies) versus coping incongruence (i.e. coping strategies that oppose each other) on a daily level (Berg and Upchurch, 2007). Lastly, if dyadic coping strategies span from underinvolvement to overinvolvement, it would be informative to capture the reasons behind caregivers’ changes between different strategies. For example, perceived partner responsiveness which was found a fundamental mediator in communication processes (Reis et al., 2004) should be investigated as a potential mechanism by which caregivers switch from one strategy to another.

The current study has important clinical implications. First, interventions that have been developed to enhance caregivers’ outcomes and optimize their role should consider how caregivers perceive their own involvement while providing care. By doing so, psychologists might assist caregivers in making collaborative and supportive efforts week by week, for example, by training them in increasing communication, collaboration skills and problem-solving with the care recipients (Northouse et al., 2010). Although caregiver-focused interventions (e.g. psychoeducational, psychotherapeutic) can be effective (Sörensen et al., 2002), the present findings suggest that also dyadic programmes might help caregivers to remain willing to care and perceive less burden (Revenson et al., 2016). Moreover, given that our results did not vary based on the type of relationship, neither on an individual-difference level (between) or on a weekly level (within), perhaps existing couples’ interventions (e.g. COCT or CCET; Bodenmann and Shantinath, 2004) can be implemented in dyads other than couples, such as older parents and adult children, siblings, friends.

The strengths of this study include its intensive longitudinal design allowing the analysis of weekly within-person processes, as well as between-person differences, and its large and multinational sample of caregivers. Moreover, having more time points per person (i.e. up to 24 consecutive questionnaires) increased the power of the study and allowed us to explore caregiving experiences over time in ways that would not be possible with other designs (Bolger and Laurenceau, 2013). Further, the results of this study added information to an emerging literature that so far has emphasized the implications of dyadic coping for care recipients but has largely neglected implications for caregivers. Lastly, age, gender and other confounding variables (e.g. care recipient’s illness, education, relationship status) were additionally analysed as covariates, and they did not alter the study results.

The results of the study should be interpreted in light of potential limitations. Given that dyadic coping was assessed by asking to evaluate strategies used ‘this week’, recall bias might still be present in the dyadic coping measures. Another limitation is given by the absence of the care recipients’ perspectives, possibly confirming caregivers’ coping strategies. Additional dyadic daily designs may shed light on whether there is an agreement between caregivers’ and care recipients’ reports of collaborative and supportive coping (Berg et al., 2008a). Finally, another limitation is that the directionality of the association between dyadic coping strategies and willingness to care and burden could not be clearly investigated as willingness and burden could also be predictors of dyadic coping strategies.

This study shed light on which dyadic coping strategies might determine *why* caregivers remain willing to care for their care recipients and are less burdened by their caring role. Clinicians should be aware that dyadic interactions and different levels of caregivers’ involvement are linked to increases or decreases in willingness to care and burden. Supporting caregivers and encouraging them to engage in collaborative and supportive behaviours towards their care recipients, independently of their relationship type, can foster caregivers’ willingness and mitigate their burden over time.

## Supplemental Material

sj-docx-1-hpq-10.1177_13591053231223838 – Supplemental material for The associations of dyadic coping strategies with caregivers’ willingness to care and burden: A weekly diary study Supplemental material, sj-docx-1-hpq-10.1177_13591053231223838 for The associations of dyadic coping strategies with caregivers’ willingness to care and burden: A weekly diary study by Giulia Ferraris, Pierre Gérain, Mikołaj Zarzycki, Saif Elayan, Val Morrison, Robbert Sanderman and Mariët Hagedoorn in Journal of Health Psychology

sj-docx-2-hpq-10.1177_13591053231223838 – Supplemental material for The associations of dyadic coping strategies with caregivers’ willingness to care and burden: A weekly diary study Supplemental material, sj-docx-2-hpq-10.1177_13591053231223838 for The associations of dyadic coping strategies with caregivers’ willingness to care and burden: A weekly diary study by Giulia Ferraris, Pierre Gérain, Mikołaj Zarzycki, Saif Elayan, Val Morrison, Robbert Sanderman and Mariët Hagedoorn in Journal of Health Psychology

sj-docx-3-hpq-10.1177_13591053231223838 – Supplemental material for The associations of dyadic coping strategies with caregivers’ willingness to care and burden: A weekly diary study Supplemental material, sj-docx-3-hpq-10.1177_13591053231223838 for The associations of dyadic coping strategies with caregivers’ willingness to care and burden: A weekly diary study by Giulia Ferraris, Pierre Gérain, Mikołaj Zarzycki, Saif Elayan, Val Morrison, Robbert Sanderman and Mariët Hagedoorn in Journal of Health Psychology
