# Supplementary material for: The associations of dyadic coping strategies with caregivers’ willingness to care and burden: A weekly diary study
Source: J Health Psychol. 2024 Jan 10;29(9):935–49. doi: 10.1177/13591053231223838 (PMC11301962; doi:10.1177/13591053231223838)
Supplement: sj-docx-1-hpq-10.1177_13591053231223838 – Supplemental material for The associations of dyadic coping strategies with caregivers’ willingness to care and burden: A weekly diary study [file sj-docx-1-hpq-10.1177_13591053231223838.docx]

Table S1

*Descriptive statistics and between-person correlations of weekly diary measures*

|  |  |  |  |  | Pearson correlations | | | | | |
| --- | --- | --- | --- | --- | --- | --- | --- | --- | --- | --- |
| Weekly diary predictors | *M (SD)* | *ICC* | *R_KRN_* | *R_CN_* | 1 | 2 | 3 | 4 | 5 | 6 |
| 1. Collaborative DC | 2.32 (.30) | 0.72 | 0.98 | 0.65 | - | .40** | -.24** | -.07** | .30** | -.29** |
| 2. Supportive DC | 3.72 (1.07) | 0.60 | 0.96 | 0.63 |  | - | -.21** | .37** | .31** | -.01** |
| 3. Uninvolved DC | 1.65 (.22) | 0.55 | 0.97 | 0.55 |  |  | - | .07** | -.45** | .33** |
| 4. Controlling DC | 3.17 (.32) | 0.72 | 0.97 | 0.47 |  |  |  | - | .04** | .31** |
| Weekly diary outcomes |  |  |  |  |  |  |  |  |  |  |
| 5. Willingness to care  (range 1-10) | 8.37 (.79) | 0.68 | 0.96 | 0.72 |  |  |  |  | - | -.39** |
| 6. Burden (range 1-5) | 2.82 (.23) | 0.65 | 0.95 | 0.68 |  |  |  |  |  | - |

*Note. ** p* < .001; DC = dyadic coping; M (SD) = means and between-person standard deviations; ICC = intraclass correlation coefficient with ICC below 0.10 as small, between 0.10 and 0.30 as medium, and above 0.30 as large, indicating the magnitude of between-group differences in the data; R_KRN_ = the between person reliability or generalizability of between person differences averaged over time; R_CN_ = the within person reliability or generalizability of within person variations averaged over items; Pearson correlation = average correlation between variables over time between-person
