# Supplementary material for: The associations of dyadic coping strategies with caregivers’ willingness to care and burden: A weekly diary study
Source: J Health Psychol. 2024 Jan 10;29(9):935–49. doi: 10.1177/13591053231223838 (PMC11301962; doi:10.1177/13591053231223838)
Supplement: sj-docx-2-hpq-10.1177_13591053231223838 – Supplemental material for The associations of dyadic coping strategies with caregivers’ willingness to care and burden: A weekly diary study [file sj-docx-2-hpq-10.1177_13591053231223838.docx]

Table S2

*Cronbach alphas at each measurement point per each dyadic coping strategies*
