# Supplementary material for: The associations of dyadic coping strategies with caregivers’ willingness to care and burden: A weekly diary study
Source: J Health Psychol. 2024 Jan 10;29(9):935–49. doi: 10.1177/13591053231223838 (PMC11301962; doi:10.1177/13591053231223838)
Supplement: sj-docx-3-hpq-10.1177_13591053231223838 – Supplemental material for The associations of dyadic coping strategies with caregivers’ willingness to care and burden: A weekly diary study [file sj-docx-3-hpq-10.1177_13591053231223838.docx]

Table S3

*Supplementary table with relationship type interaction term*

|  | Willingness to care | | | | Burden | | | | | |
| --- | --- | --- | --- | --- | --- | --- | --- | --- | --- | --- |
|  |  |  |  | CI_95%_ | |  | |  | CI_95%_ | |
| Fixed effects (intercept, slopes) | *b (SE)* | *t _(954)_* | *p* | Lower | Upper | *b (SE)* | *t _(954)_* | *p* | Lower | Upper |
| Intercept | 8.68 (.10) | 86.58 | <.001 | 8.49 | 8.88 | 2.84 (.05) | 56.15 | <.001 | 2.74 | 2.94 |
| Time slope (week 0-23) | -0.65 (.06) | -10.05 | <.001 | -0.78 | -0.52 | 0.05 (.03) | 1.66 | .096 | -0.01 | 0.12 |
| Between-person associations | | | | | | | | | | |
| Collaborative DC *relationship type | -0.13 (.14) | -0.93 | .352 | -0.41 | 0.14 | 0.15 (.07) | 2.07 | .380 | 0.00 | 0.30 |
| Supportive DC *relationship type | -0.16 (.20) | -0.817 | .414 | -0.55 | 0.22 | -0.08 (.10) | -0.84 | .396 | -0.29 | 0.11 |
| Uninvolved DC *relationship type | -0.12 (.19) | -0.65 | .515 | -0.51 | 0.25 | 0.19 (.10) | 1.91 | .559 | -0.00 | 0.39 |
| Controlling DC *relationship type | 0.17 (.15) | 1.13 | .256 | -0.12 | 0.46 | 0.04 (.07) | 0.54 | .589 | -0.11 | 0.19 |
| Within-person associations | | | | | | | | | | |
| Relationship type (spousal vs. non-spousal) | -0.27 (.12) | -2.19 | .229 | -0.52 | -0.02 | -0.03 (.06) | -0.57 | .565 | -0.16 | 0.08 |
| Collaborative DC *relationship type | 0.01 (.06) | 0.25 | .799 | -0.10 | 0.13 | 0.00 (.03) | 0.17 | .859 | -0.06 | 0.07 |
| Supportive DC *relationship type | 0.06 (.07) | 0.85 | .392 | -0.07 | 0.20 | -0.00 (.03) | -0.05 | .958 | -0.07 | 0.07 |
| Uninvolved DC *relationship type | 0.09 (.08) | 1.18 | .237 | -0.06 | 0.26 | -0.05 (.03) | -1.33 | .184 | -0.13 | 0.02 |
| Controlling DC *relationship type | 0.01 (.06) | 0.16 | .866 | -0.11 | 0.13 | -0.02 (.03) | -0.62 | .533 | -0.09 | 0.04 |
